# Life in a time of COVID: a mixed method study of the changes in lifestyle, mental and psychosocial health during and after lockdown in Western Australians

**DOI:** 10.1186/s12889-021-11971-7

**Published:** 2021-10-26

**Authors:** Ranila Bhoyroo, Paola Chivers, Lynne Millar, Caroline Bulsara, Ben Piggott, Michelle Lambert, Jim Codde

**Affiliations:** 1 Institute for Health Research, The University of Notre Dame Australia, 19 Mouat Street, PO Box 1225, Fremantle, Western Australia 6959 Australia; 2 School of Population Health, Curtin University, Perth, Australia; 3 School of Health Sciences, The University of Notre Dame Australia, Perth, Australia; 4 Disciplines of Psychology and Exercise Science, Murdoch University, Perth, Australia; 5 School of Medical and Health Sciences, Edith Cowan University, Perth, Australia; 6 Injury Matters, Perth, Australia; 7 Division of Obstetrics & Gynaecology, The University of Western Australia, Perth, Australia

**Keywords:** Depression, Stress, Loneliness, Nutrition, Social isolation, Physical activity, COVID-19, Pandemic, Health promotion

## Abstract

**Background:**

Since the beginning of the COVID-19 pandemic, the Western Australian government imposed multiple restrictions that impacted daily life activities and the social life. The aim of this study was to examine the effects of COVID-19 lockdown on the community’s physical, mental and psychosocial health.

**Methods:**

Approximately 2 months after a three-month lockdown, a cross-sectional study was opened to Western Australian adults for an 8-week period (25th August – 21 October 2020). Participants competed a 25-min questionnaire adapted from the Western Australia Health and Wellbeing Surveillance system. Participants provided information on their socio-demographic status, lifestyle behaviours, mental health, and psychosocial health during and post-lockdown. Open-ended questions explored key issues in greater detail. Changes between the lockdown and post-lockdown period were assessed using Wilcoxon signed rank test and One-Sample Kolmogorov-Smirnov Normal tests as appropriate. Sex differences were examined using the Mann-Whitney U test. A content analysis approach examined responses to the open-ended questions with frequencies and variations in responses determined using Chi-Square tests.

**Results:**

A total of 547 complete responses were obtained. Compared to post-lockdown period, lockdown was associated with a significantly lower levels of physical activity, poorer mental well-being and sense of control over one’s life, and a higher level of loneliness. Similarly, during lockdown, there was a significantly higher consumption of junk food, soft drinks and alcoholic drinks but no change in fruit and vegetable intake. Participants recalled health campaigns on hand washing and social distancing and there was a retrospective view that more timely and informative campaigns on physical activity, nutrition and mental well-being should have been available during lockdown.

**Conclusions:**

While advice on infection control measures were appropriately provided, there is a need for concurrent health promotional information to help combat the changes in physical, mental and psychosocial well-being observed during quarantine to prevent negative health consequences in the community even if there are minimal effects of the pandemic itself.

**Supplementary Information:**

The online version contains supplementary material available at 10.1186/s12889-021-11971-7.

## Introduction

Since the first reports of a novel coronavirus (COVID-19) in late 2019, the spread of this virus rapidly progressed into a global pandemic. By May 2021, almost 142 million people had become infected with over 3 million associated deaths [1]. By contrast, the Western Australian community of 2.6 million people had fewer than 1000 infections (most acquired while travelling overseas) and only nine deaths [2].

One of the government’s key strategies in minimising the impact of COVID-19 was to rapidly close the national and Western Australia state boarders to travellers in March 2020. The state government also implemented additional restrictions involving the movement of people within the community, which at the highest level of restrictions, included permit-only regional intra-state travel; closure of social venues such as bars, clubs, sporting venues, cinemas, cultural institutions, places of worship; restaurants and cafes were limited to takeaway services; schools were closed; work from home arrangements for many businesses; and weddings, funerals and household visitors were limited to small numbers. In the following months, from mid-May through to late June 2020, the government gradually eased many of these restrictions. As of March 2021, while some restrictions remained in place, the community was operating at some level of normality [3].

A recent systematic review confirmed that quarantine confinement due to infectious disease outbreaks can result in negative psychological outcomes, including insomnia, depression, anxiety, post-traumatic stress, and emotional exhaustion [4]. Similarly, it has been shown that quarantine and isolation can have psychological effects impacting overall lifestyle, level of physical activity and can potentially lead to unhealthy consumption of certain foods and alcohol, all of which carry some long-term effects on cardiovascular disease [5]. Further research reported that sedentarism during home confinement can create detectable changes in muscle wastage within 2 days and that positive energy balance during physical inactivity is linked with fat deposition, associated with systemic inflammation and activation of antioxidant defences, exacerbating muscle loss [5]. Perhaps more importantly, these deleterious effects of inactivity could be diminished by routine exercise practice, although the exercise dose response relationship is currently unknown in this scenario [6]. Numerous studies have reported on the importance of social relationships on healthy nutritional behaviours [7, 8] that bode poorly for periods of social distancing and isolation. In their brief paper titled ‘Nutritional recommendations for COVID-19 quarantine’, the authors describe that quarantine is associated with the interruption of the work routine that can lead to boredom which in turn has been associated with a greater energy intake, as well as the consumption of higher quantities of fats, carbohydrates, and proteins with the associated stresses, pushing people toward overeating, mostly of sugary *comfort foods* [9]*.* This is of concern as a dietary pattern characterised by higher intake of processed and unhealthy foods has been shown to be associated with an increased likelihood of higher psychological symptomatology and clinical depression [10, 11].

A growing number of studies have emerged from the COVID-19 period. For example, a large survey conducted in China reported higher prevalence of anxiety and depression in the general population during lockdown [12]. Similarly, a study of non-infected people in both Italy and Israel found higher levels of anxiety and depression during the COVID-19 pandemic compared to the reported prevalence in the general population in previous years [13]. In a survey of Australian adults, Stanton and colleagues reported negative changes in physical activity, sleep, smoking and alcohol intake were associated with higher depression, anxiety and stress symptoms since the onset of COVID-19 [14].

With calls for effective health promotion strategies directed at adopting or maintaining positive health-related behaviours [15–17], the aim of this study was to provide a cross-sectional analysis of the impact of COVID-19 on the wider community’s physical, mental and psychosocial health and to identify issues that may persevere after the restrictive measures have been lifted. Particularly to the present study, the term ‘psychosocial health’ was used as another dimension of health and well-being other than just physical and mental health. This includes level of control, loneliness and resilience to factors that impact on a person’s life. The results will help inform current and future health promotion strategies to better assist people maintain their quality of life and healthy lifestyle.

## Methods

### Study design

Following a request from the state government to rapidly identify the impact of lockdown on the community to inform any future restrictions imposed due to the pandemic, Western Australian residents aged 18 years or older were invited to complete a cross-sectional study using a web-based survey. The survey was open for an 8-week period (between 25th August – 21st October 2020) and commenced approximately 2 months after an enforced community lockdown of 3 months duration (23rd March – 27th June 2020). A mixed method approach using the triangulation design was used.

A priori sample size calculation was conducted based on between group (e.g., five age groups) differences using a medium effect size of 0.31, *α* = 0.05, and a power = 0.80 which indicated the need to recruit a minimum sample of 174 participants. This sample size was determined a priori to also enable lockdown and post-lockdown response comparisons (G*Power version 3.1.9.2; 2014).

The survey was promoted through a range of state and local community newspapers and a variety of social media outlets and snowballed through professional linkages. Ethical approval was granted by the University of Notre Dame Human Research Ethics Committee (HREC, 2020-133F).

### Questionnaire design

Participants completed the questionnaire using an online platform (Qualtrics, Provo, UT). The initial page of the survey presented, every potential respondent with an information page that outlined the purpose of the study, estimated duration (25 mins) and anonymity of survey responses. In accordance with HREC requirements, respondents confirmed their consent for their data to be used in the study.

As the main aim of the study was to explore differences in physical, mental and psychosocial behaviours during COVID-19 lockdown restrictions and the post-lockdown period, participants were asked to respond to the questions reflecting on both time periods. As a reminder, a list of the lockdown restrictions was provided at the start of the survey and then throughout the survey in the form of a pop-up window to assist recollection.

The survey comprised of questions regarding their basic socio-demographic information, lifestyle behaviours, mental health, and psychosocial health. In addition, several open-ended questions explored these key issues in more detail.

#### Socio-demographic information

This series of questions gathered information about the participants that included sex (Male, Female, Other), age (years), locality (Urban vs Rural), level of education (Degree or higher, Diploma or Trade, High School, Did not complete High School), employment status (Employed, Unemployed, Student, Retired, other), and Household income (<$40,000, $40,000–$100,000, >$100,000).

#### Lifestyle behaviours

Questions about lifestyle behaviours explored physical activity, sedentary behaviours, nutrition, and alcohol use and were adopted from the Western Australia Health and Wellbeing Surveillance system (WA-HWSS) survey [18].

#### Mental health

Mental health was assessed using DASS-21 [19]. DASS-21 measures depression, anxiety and stress during the last week via a 21-item questionnaire. Each item is a statement about a negative emotional symptom and is rated by the participant on a 0–3 scale based on the extent to which they experienced that symptom over the past week. The scores for each of the three scales (i.e., depression, anxiety, and stress) are derived by summing the scores for the seven items that comprise that scale and multiplying the sum by 2. Higher scores reflect increased severity of the measures. The total score for each scale is converted to a classification (i.e., ‘Normal’, ‘Mild’, ‘Moderate’, ‘Severe/Extremely severe’) using the DASS Manual [19]. Internal consistency (Cronbach’s alpha) is high for each scale (depression = 0.94, anxiety = 0.87, Stress = 0.91) [20].

Kessler-10 (K-10) is a global measure of psychological stress [21]. It is a 10-item questionnaire scored on a five-point Likert scale resulting in a cumulative score of 10 to 50 which are categorised as ‘Likely to be well’ [10–19], ‘Likely to have a mild mental disorder’ [20–24], ‘Likely to have a moderate mental disorder’ [25–29] and ‘Likely to have a several mental disorder’ [30–50]. The K-10 is a valid and reliable instrument for screening psychological distress at a population level [21]. It is used by Australian Bureau of Statistics as part of their regular data collections [22] and been found to outperform other screening tools amongst an Australian population [23].

#### Psychosocial health

The level of perceived lack of control and loneliness were used as measures of psychosocial health. Perceived lack of control was measured using three items adopted from the WA-HWSS survey. The questions were ‘*How much of the time did you feel a lack of control over your life in general*’, ‘*How much of the time did you feel a lack of control over your personal life’* and ‘*How much of the time did you feel a lack of control over your health*’, all of which captured the response using a five-point Likert scale as ‘Always’, ‘Often’, ‘Sometimes’, ‘Rarely’, or ‘Never’.

Loneliness was measured using the validated UCLA three-item scale with responses ‘Hardly ever’, ‘Some of the time’, and ‘Often’ to 12 questions that include ‘*How often do you feel you lack companionship?*’, ‘*How often do you feel left out?*’, and ‘*How often you feel isolated from others?*’ [24]. The UCLA-3 has a reported alpha coefficient of reliability of 0.72 and the internal consistency for a three-item scale was quite good indicating that the items reliably measure loneliness. The questions were scored 1 to 3, then summed to a score ranging from 3 to 9. Loneliness was subsequently categorized as follows: ‘No loneliness’ (3, 4), ‘Moderate loneliness’ (5–7), and ‘Severe loneliness’ (8, 9) [25].

#### Open ended questions

In addition to the Likert style survey questions, the participants were asked a series of open-ended question about several topics to explore their responses in greater detail (see Supplementary information). Examples include:

- Thinking back to COVID-19 lockdown, what would you say was the biggest difference made to your physical activity (in other words, any changes to your physical activity preferences, types of physical activity, physical activity intensity, etc.)? Please describe those changes.
- Thinking back to COVID-19 lockdown, what would you say had been the biggest difference you made to your diet (in other words, any changes to your food preferences, types of food, food preparation, cooking, alcohol intake etc.)? Please describe what changed.
- Describe what may have affected (positively and/or negatively) your mental well-being during the COVID-19 lockdown period?

Similarly, the participants were asked three questions regarding their views on the government’s health promotion campaign: (a) what health promotion and safety campaigns they recalled from the COVID-19 lockdown period, (b) what personal changes they made in response to these messages and (c) what they felt would be useful in the advent of another lockdown.

### Data analysis

Sociodemographic and survey data were summarised as percentages (%). As the survey data scores were not normally distributed (Shapiro-Wilk test), group differences in lifestyle behaviours, mental health and psychosocial health for lockdown/post-lockdown periods for all respondents, and males and females separately, was examined using Wilcoxon signed rank test for paired responses.

As most questions required a Likert response, relative changes between the two time periods were calculated to produce a new 3-point measure that indicated whether the response during the lockdown period was ‘Increased’, ‘Same’ or ‘Decreased’ compared to the post-lockdown period. This allowed assessment of the impact of the lockdown regardless of the individual’s baseline measure. One-Sample Kolmogorov-Smirnov Normal Tests were conducted to assess whether there was an asymmetrical shift in the behaviours between the two periods at the population and sex levels.

No respondents identified sex as ‘other’ therefore differences between male and female responses were compared using the Mann-Whitney U test. Statistical analysis was performed using SPSS version 26.0 (IBM, Chicago, IL, USA) with results considered statistically significant for *p* value < 0.05. Cohen’s d (d) effect sizes were calculated from the Z-statistic generated from the Wilcoxon, Kolmogorov-Smirnov or Mann-Whitney test using an online calculator [26].

Quantitative content analysis was undertaken for the open-ended question responses with up to three unique points per question being extracted from each respondent. The exact wording of the extracted points was themed and assigned a numerical code to enable frequency analysis based on the multiple responses to each question [27]. This was descriptively described by simple frequency analysis and then variations between sub-populations of the study population determined by Chi-square analysis.

## Results

### Participants

A total of 793 people indicated their consent to participate in the study. Following exclusion of those participants who were not residents of Western Australia and those who provided consent to participate but did not respond to any questions, 547 participants (69%) were included in the study. As only one participant reported testing positive for COVID-19, they were removed from this study analysis resulting in a final sample of 546 respondents. As summarized in Table 1, the majority of respondent were aged 45 years or over, female, university educated, lived within the metropolitan area, employed, and had a household income of at least $100,000.

**Table 1:** Socio-demographic information of participants

Characteristics	%
**Age** (546)	
18–24	11.9
25–34	13.6
35–44	14.8
45–54	20.5
55–64	21.1
65+	18.1
**Sex** (540)	
Male	25.4
Female	74.6
**Residential location** (544)	
Urban	87.7
Rural	12.3
**Education** (294)	
Bachelor degree or higher	69.7
Diploma or certificate in Trade/apprenticeship	18.0
Completed high school	10.9
Did not complete high school	1.4
**Household income** (265)	
Under $40,000	12.5
$40,000–$100,000	37.0
$100,000 and above	50.6
**Employment during lockdown** (291)	
Employed (Inc self-employment)	64.9
Unemployed	6.9
Student	6.9
Retired	17.2
Other	4.1

*Note.* The number of respondents (*n*) for each characteristic is provided in brackets

### Changes in physical activity

Levels of physical activity during the lockdown period (65%) was significantly lower compared to the post-lockdown (78%) period and this was consistent for both males and females (Table 2). While there was an overall shift to lower levels of physical activity during lockdown, this finding was not universal. One-third (34%) of respondents reduced their activity during lockdown, but 15% reported increased activity (Table 3). This effect was similar in both sexes. The reasons underpinning this diversity of response are reflected in the responses to the question “*What would you say was the biggest difference made to your physical activity?”* (Table 4). The need to change their physical activity routines was the most reported impact of the lockdown.

*Change of activity from swimming to the walking. (23 yo male)*

Compared to those who decreased their level of physical activity during lockdown, many that reported increased level of physical activity felt they had more time to do so (*χ*^*2*^ = 69.33, *p* < 0.001, *n* = 391, *d* = 0.93) and that it helped their mental health (*χ*^*2*^ = 22.45, *p* < 0.001, *n* = 391, *d* = 0.49):

*Not having the commute to work in the morning meant I had an hour to do home workout using a fitness app before work, without having to wake up any earlier. (25 yo female)*

For others, working from home reduced their usual level of physical activity:

*As I was working from home, I was sitting more as opposed to being active pre-covid (going up and down stairs, taking public transport, walking). Sitting more translated to being less active. (33 yo female)*

Those who decreased their physical activity often did so because of limited options, fear of increased risk of infection, reduced team sports activity, and inability to remain motivated.

*I didn’t feel I had the desire to exercise while my yoga studio was closed. There was too much panic around cleanliness and distancing that I felt it best to stay home. (29 yo female)*

*Lack of motivation. Feeling down. Watching too much tv. (60 yo female)*

**Table 2:** Self-reported level of physical activity during and after the lockdown

	Persons				Males				Females			
	Lockdown		post-lockdown		Lockdown		post-lockdown		Lockdown		post-lockdown	
	***n***	%	***n***	%	***n***	%	***n***	%	***n***	%	***n***	%
Very active	40	8.8	55	12.0	9	8.0	16	14.0	31	9.1	39	11.3
Active	114	25.1	130	28.3	32	28.6	38	33.3	82	24.0	92	26.6
Moderately active	141	31.1	175	38.0	31	27.7	36	31.6	110	32.2	139	40.2
Not very active	119	26.2	83	18.0	27	24.1	22	19.3	92	26.9	61	17.6
Not active at all	40	8.8	17	3.7	13	11.6	2	1.8	27	7.9	15	4.3
Total	454	100.0	460	100.0	112	100.0	114	100.0	342	100.0	346	100.0
Wilcoxon sign ranked test (*Z*)	−5.48***, *d* = 0.37				−3.56***, *d* = 0.49				−4.18***, *d* = 0.32			

*Note.* *** = *p*-value < 0.001; *d* = Cohen’s D

**Table 3:** Relative change in physical activity during lockdown compared to the post-lockdown period

	Persons		Males		Females	
	***n***	%	***n***	%	***n***	%
Increase	70	15.4	14	12.5	56	16.4
Same	229	50.4	57	50.9	172	50.3
Decrease	155	34.1	41	36.6	114	33.3
Total	454	100.0	112	100.0	342	100.0
Kolmogorov-Smirnov Test (*t*)	*t* = 0.27***		*t* = 0.28***		*t* = 0.26***	

*Note.* *** = *p*-value < 0.001

**Table 4:** How lockdown impacted on physical activity by reported change in physical activity levels

	Relative change in physical activity (%)			
Coded responses	Increased (***n*** = 89)	Same (***n*** = 267)	Decreased (***n*** = 202)	Total (***n*** = 562)
Routine changed	45.8	33.1	21.5	31.1
More sedentary	6.8	13.8	36.2	20.4
Limited options	1.7	18.8	27.7	19.0
No change	8.5	25.4	6.2	16.1
Had more time for PA	33.9	9.9	0.8	10.5
Substitutes for gym	3.4	6.6	10.8	7.5
Exercised at home	8.5	6.6	6.2	6.7
Fear of infection reduced PA	–	5.0	8.5	5.4
Made more effort PA	3.4	3.9	6.9	5.1
Social distancing impacted on PA	15.3	5.5	–	5.1
Online exercise videos	5.1	3.3	5.4	4.6
Reduced team sports	–	4.4	4.6	3.8
PA helped with MH	13.6	1.7	0.8	3.2
Reduced PA over time period	1.7	1.1	6.2	2.9
Job loss reduced PA	–	0.6	3.1	1.3
More infection awareness from PA	–	0.6	2.3	1.1
Other	3.4	7.2	8.5	7.0

*Note.* Percentages (%) are based on multiple responses rather than persons

### Changes in food and alcohol consumption

Consumption of fruit and vegetables remained similar during the lockdown and post-lockdown periods. Of note, the consumption of grain-based products (e.g., bread, cereal and pasta), snack meals, soft drinks and alcohol were all significantly higher during the lockdown period (Table 5). Sub-analysis by sex revealed similar changes in the female respondents with an increase in consumption of grain consumption (*Z* = − 5.24, *p* < 0.001, *d* = 0.45), soft drink (number of days per week: *Z* = 3.16, *p* = 0.002, *d* = 0.26; number of cups per day: *Z* = − 3.16, *p* = 0.002, *d* = 0.27), take away snack meals (*Z* = − 2.46, *p* = 0.014, *d* = 0.36) and alcohol (number of standard drinks per day: *Z* = 2.90, *p* = 0.004, *d* = 0.25). For males, a significant increase was reported in the number of times per week they purchased snack foods (pies, burgers, pizza, chicken, or chips from places like McDonalds, Hungry Jacks, Pizza Hut or Red Rooster; *Z* = − 2.47, *p* = 0.014, *d* = 0.38) and the number of days per week they consumed alcohol (*Z* = − 3.12, *p* = 0.002, *d* = 0.49). Comparison between the sexes only revealed a difference in grain consumption (*Z* = 2.93, *p* = 0.003, *d* = 0.22).

**Table 5:** Changes in food and alcohol consumption during lockdown relative to post-lockdown period

	Increased (%)	Same (%)	Decreased (%)	Wilcoxon Sign Rank (***Z***)	Cohen’s ***d***
**Daily number of serves** (373)					
Vegetables	12.6	74.5	12.9	− 0.24	0.02
Fruit	14.2	73.2	12.6	−0.39	0.04
Grain	22.0	69.4	8.6	−4.83***	0.36
Meat	13.1	78.8	8.0	−1.70	0.18
**Snack meals per week** (372)	20.4	66.7	12.9	− 3.35***	0.25
**Consumption of soft drinks**					
Days per week (389)	11.6	84.1	4.4	−3.37***	0.24
Cups per day (130)	23.1	67.7	9.2	3.35***	0.24
**Alcohol consumption**					
Days per week (370)	35.1	55.9	8.9	−8.31***	0.64
Standard drinks per day (363)	23.1	66.4	10.5	−3.08**	0.23

*Note.* Statistical analysis was performed using Wilcoxon Sign Ranked test on unaggregated response data. ** = *p*-value < 0.01. *** = *p*-value < 0.001. The number of respondents (*n*) for each item are provided in brackets

Responses most frequently reported to the question “*What would you say was the biggest difference you made to your diet (in other words, any changes to your food preferences, types of food, food preparation, cooking, alcohol intake etc.) during the lockdown*?” and “*What would you say had been the biggest difference you made to your dining practices (e.g., less dining out, more take away food, more food deliveries, etc.)?”* are shown in Table 6.

**Table 6:** Responses most frequently reported to open-ended questions about the impact of lockdown on dietary behaviours

	Persons (%)	Males (%)	Females (%)
***What were the biggest differences to your diet during lockdown?***			
*n*	*466*	*105*	*359*
No changes	26.8	40.0	22.7
Cooked more	23.9	14.1	27.1
Ate less healthy food	18.6	21.2	17.8
Drank more alcohol	16.1	12.9	17.1
Ate healthier food	13.5	12.9	13.4
Consumed more food	12.7	3.5	15.6
Had more takeaway	7.6	7.1	7.8
Ate less food	5.9	7.1	5.6
Drank less alcohol	3.1	1.2	3.7
Other	3.1	3.6	2.6
***What were the biggest differences to your dining practices during lockdown?***			
*n*	*487*	*112*	*373*
Ate out less	48.3	49.4	48.1
More takeaway	28.4	32.1	27.4
More cooking at home	16.8	9.9	18.9
No difference	16.5	23.5	14.4
More food deliveries	8.8	3.7	10.4
Less deliveries due to COVID fear	6.3	6.2	6.3
Eat healthier	4.0	–	5.2
More unhealthy food	2.6	3.7	1.9
More alcohol	1.4	1.2	1.1
Weight gain	1.4	1.2	1.5
Other	4.0	7.4	3.0

*Note.* Percentages (%) are based on number of responses

Approximately a quarter of the responses suggested that the lockdown period had little impact on their diet although this was more common in males than females (*χ*^*2*^ = 12.40, *p* < 0.001, *d* = 0.34).

*No real change to diet, although I tended to cook more elaborate meals due to having more time. (29 yo male).*

Many people reported cooking more often (*χ*^*2*^ = 7.48, *p* = 0.006, *d* = 0.26). Many also reported eating less healthy food with a similar number saying they drank more alcohol, with responses from males and females not detected to be significantly different.

*I cooked more comfort food and large meals I could freeze and starting drinking alcohol earlier in the afternoon than before Covid-19. (71 yo female)*

*More fast food, less home cooked meals. Higher preference for unhealthy foods. (23 yo male)*

In response to the question “*What would you say was the biggest difference you made to your dining practice during the lockdown*?”, most people replied that they ‘ate out less’ (48%) followed by ‘more take away food’ (28%) and more ‘home delivery’ (9%). However, as highlighted by the quotes below, the reasons underpinning this change in behaviour varied:

*Less dining out... More take away food for the first few months as we wanted to support local businesses however this got quite expensive quickly. (28 yo female)*

*We avoided supermarkets and enjoyed home delivery by Woolworths. We ceased dining out at restaurants and with friends. (71 yo male)*

*We got hello fresh delivered during lockdown which we did have before sometimes, but we choose it because we didn’t want to go the grocery store and it was easy and healthy. We are out less because of the restaurants closing. (23 yo female)*

Almost a fifth of respondents indicated they cooked more at home as a result of the lockdown although this was more commonly reported by females (χ^2^ = 6.60, *p* = 0.010, d = 0.24).

*I used to dine out at least once a week but that stopped when restaurants closed. I cooked a lot more than I had previously and also got food deliveries. I started to get a bit more creative in the kitchen due to some food shortages when people were stockpiling. (31 yo female)*

*I cooked a lot more roasts as I was home all day and could better monitor the oven. (27 yo female)*

The proportion of people who reported increased food deliveries (8.8%) was similar to those who claimed to reduce their number of deliveries due to fear of COVID-19 (6.3%).

*More deliveries. More take out. (57 yo female)*

*We didn't want to risk take-away food so had none. (53 yo female)*

### Changes in mental health and psychosocial health

Lockdown was associated with a significant increase in self-reported mental disorders with 40% of respondents reporting mild to severe mental disorders (Kessler-10) compared to 28% in the post-lockdown period. This effect was observed in both males and females (Table 7). A significant increase in the levels of depression and stress were identified using the DASS-21 instrument, but not for anxiety. At the sex level, only females showed a statistically significant increase in the level of depression across the two time periods (Table 8).

**Table 7:** Presence of psychological distress using the Kessler Psychological Distress Scale (K10) for persons, males and females

	Persons				Males				Females			
	Lockdown		Post-lockdown		Lockdown		Post-lockdown		Lockdown		Post-lockdown	
	***n***	%	***n***	%	***n***	%	***n***	%	***n***	%	***n***	%
Likely to be well	208	60.5	254	72.0	57	67.9	66	78.6	151	58.3	188	70.1
Moderate distress	50	14.5	39	11.0	13	15.5	6	7.1	36	13.9	32	11.9
Mild mental disorder	36	10.5	22	6.2	5	6.0	8	9.5	31	12.0	14	5.2
Severe mental disorder	50	14.5	38	10.8	9	10.7	4	4.8	41	15.8	34	12.7
Total	344	100.0	353	100.0	84	100.0	84	100.0	259	100.0	268	100.0
Wilcoxon sign rank (*Z*)	−5.21***, *d* = 0.41				−2.98**, *d* = 0.47				−4.49***, *d* = 0.39			

*Note.* ** = *p*-value < 0.01; *** = *p*-value < 0.001; *d* = Cohen’s D

**Table 8:** Presence of Depression, Anxiety and Stress Scale (DASS-21) for persons, males and females

	Depression				Anxiety				Stress			
	Lockdown		Post-lockdown		Lockdown		Post-lockdown		Lockdown		Post-lockdown	
	%	***n***	%	***n***	%	***n***	%	***n***	%	***n***	%	***n***
**Persons**												
Normal	193	65.0	216	72.2	237	80.1	246	82.0	226	80.7	252	85.1
Mild	32	10.8	30	10.0	16	5.4	19	6.3	20	7.1	15	5.1
Moderate	36	12.1	26	8.7	14	4.7	9	3.0	19	6.8	9	3.0
Severe	10	3.4	10	3.3	10	3.4	9	3.0	14	5.0	17	5.7
Extremely severe	26	8.8	17	5.7	19	6.4	17	5.7	1	0.4	3	1.0
Wilcoxon sign rank (*Z*)	−2.71**, *d* = 0.22				−0.98, *d* = 0.08				−1.99*, *d* = 0.17			
**Males**												
Normal	46	65.7	46	65.7	59	84.3	60	84.5	55	84.6	60	85.7
Mild	6	8.6	6	8.6	3	4.3	5	7.0	6	9.2	3	4.3
Moderate	9	12.9	9	12.9	3	4.3	1	1.4	2	3.1	3	4.3
Severe	2	2.9	2	2.9	–	0.0	1	1.4	2	3.1	3	4.3
Extremely severe	7	10.0	7	10.0	5	7.1	4	5.6	–	0.0	1	1.4
Wilcoxon sign rank (*Z*)	−1.89, *d* = 0.32				−0.30, *d* = 0.05				−0.81, *d* = 0.14			
**Females**												
Normal	146	64.6	163	71.8	177	78.7	185	81.1	170	79.4	191	84.9
Mild	26	11.5	24	10.6	13	5.8	14	6.1	14	6.5	12	5.3
Moderate	27	11.9	18	7.9	11	4.9	8	3.5	17	7.9	6	2.7
Severe	8	3.5	8	3.5	10	4.4	8	3.5	12	5.6	14	6.2
Extremely severe	19	8.4	14	6.2	14	6.2	13	5.7	1	0.5	2	0.9
Wilcoxon sign rank (*Z*)	−2.09*, *d* = 0.20				−0.87, *d* = 0.08				−1.82, *d* = 0.18			

*Note.* * = *p*-value < 0.05; ** = *p*-value < 0.01; *d* = Cohen’s D

Feedback provided to the question “*Describe what may have affected (positively and/or negatively) your mental well-being during the COVID-19 lockdown period?*” provide some insights to these changes. From a negative perspective:

*Lack of social contact, confinement at home, concern about my own health and that of family members, worry about job security, despair at the mistakes made by governments and irresponsibility, ignorance and selfishness displayed by some people, the sense that the situation was bad and made still worse by foolishness and malice. (52 yo male)*

*Being locked in a house 100% of the time with my significant other was “detrimental” to our relationship. We just kept getting on each other’s nerves. (41 yo male)*

Others reported a positive effect of the lockdown:

*Increased physical exercise indoors and outdoors, spending more time more frequently with family, and more time watching videos. I enjoyed [it] all [and it] affected my mental health positively. (30 yo male)*

During the lockdown period, significantly fewer respondents (52%) reported always or usually having control over their general lives compared to 72% in the post-lockdown period. Similar significant findings were observed in both sexes (Table 9). Control factors described included:

*Increased stress due to alarm and panic buying, and sense that society was beginning to destabilise in certain ways. Heightened alertness to the risk of contracting Covid-19. Sense of restriction on movement outside the house. Increased worry over health and future of older relatives and friends. (28 yo male)*

*Missed family milestones (eg. grandchildren's birthdays), difficult to visit mother in Aged Care (long travel time for very short visit). (68 yo female)*

Similar significant differences were also observed for the questions about level of control over one’s personal life and health for all respondents (*Z* = − 5.13, *p* < 0.001, *d* = 0.43; *Z* = − 5.81, *p* < 0.001, *d* = 0.50), for males (*Z* = − 2.67, *p* = 0.008, *d* = 0.46; *Z* = − 3.63, *p* < 0.001, *d* = 0.64), and females (*Z* = − 4.41, *p* < 0.001, *d* = 0.43; *Z* = − 4.54, *p* < 0.001, *d* = 0.44).

**Table 9:** Response summary for question: How much of the time did you feel in control over your life in general?

	Persons				Males				Females			
	Lockdown		Post-lockdown		Lockdown		Post-lockdown		Lockdown		Post-lockdown	
	***n***	%	***n***	%	***n***	%	***n***	%	***n***	%	***n***	%
Always	68	23.0	92	30.9	20	28.2	25	35.2	48	21.4	67	29.9
Often	85	28.7	122	40.9	20	28.2	26	36.6	65	29.0	96	42.9
Sometimes	76	25.7	50	16.8	17	23.9	13	18.3	58	25.9	36	16.1
Rarely	49	16.6	25	8.4	8	11.3	5	7.0	41	18.3	20	8.9
Never	18	6.1	9	3.0	6	8.5	2	2.8	12	5.4	7	3.1
Total	296	100.0	298	100.0	71	100.0	71	100.0	224	100.0	226	100.0
Wilcoxon sign rank (*Z*)	−14.91***, *d* = 1.54				−7.32***, *d* = 1.56				−12.98***, *d* = 1.55			

*Note.* *** = *p*-value < 0.001; *d* = Cohen’s D

In addition to increased sense of a lack of control over their lives, almost 70% of respondents reported moderate to severe levels of loneliness during the lockdown compared to 55% during the post-lockdown period. Similar impact was observed in both males and females (Table 10). Factors impacting this sense of loneliness is articulated below:

*Unable to meet with friends and family. Unable to visit family back in my country of origin, hearing and reading bad news about [the] pandemic, feeling lonely, working at the University campus which was deserted, no extra activities after work. (39 yo female)*

**Table 10:** Level of loneliness for persons, males and females

	Persons				Males				Females			
	Lockdown		Post-lockdown		Lockdown		Post-lockdown		Lockdown		Post-lockdown	
	***n***	%	***n***	%	***n***	%	***n***	%	***n***	%	***n***	%
Not lonely	91	30.8	135	45.2	20	28.2	35	48.6	71	31.8	100	44.2
Moderately	90	30.5	77	25.8	27	38.0	20	27.8	62	27.8	57	25.2
Severely	114	38.6	87	29.1	24	33.8	17	23.6	90	40.4	69	30.5
Total	295	100.0	299	100.0	71	100.0	72	100.0	223	100.0	226	100.0
Wilcoxon sign rank (*Z*)	−5.47***, *d* = 0.46				−3.55***, *d* = 0.62				−4.41***, *d* = 0.43			

*Note.* *** = *p*-value < 0.001; *d* = Cohen’s D

### Awareness and impact of health and safety campaigns

Analysis of the open-ended question regarding what health promotion and safety campaigns the respondent’s recalled during the COVID-19 lockdown period revealed that the most common health messages recalled involved hand hygiene and social distancing, with most frequent responses summarised in Table 11. Almost a quarter of respondents did not recall seeing any health and safety campaigns from the lockdown period. Over a third of respondents claimed that they did not change any of their personal behaviours because of the health promotional material they had observed although many reported they washed their hands, used sanitizer, and observed social distancing.

*None, we've always had clean practices. (59 yo male)*

*I stopped listening to media, government and experts as their data has been shown to constantly conflict and later turn out to be false, an over statement or misstated to the public to create fear. (45 yo male)*

In response to the question about what health promotion or safety material they would like in the advent of another outbreak, many wanted better and timelier information about the outbreak with others requesting more physical, nutritional, and mental health support information.

*I think overall the information is already out there. During the first lock down governments were struggling to organise their messages as they were naturally making it up as they went along. They are a lot better at staying on message now and talking plainly and openly about what happens. (63 yo male)*

*Just make the requirements clear and easily found. Keep them updated at the same location rather than putting changes or new requirements in announcements. (39 yo male)*

When those changes identified as significantly different between the lockdown and post-lockdown period were further analysed by those who either increased or decreased their behaviours, some significant variations were observed. For example, as shown in Table 11, differences in recall of health promotion campaigns were observed between those who increased or decreased their level of physical activity, daily alcohol intake or the number of standard drinks per day.

**Table 11:** Most frequent reported responses to open-ended questions about health promotion campaigns

	Persons (%)	Increase (%)	Decrease (%)	x^**2**^	d
**Recall of health promotion campaigns (411)**					
Washing hands	51.7				
Social distancing	39.6				
*By change in physical activity*		52.4	31.6	4.96*	0.41
*By change in daily alcohol consumption*		36.0	66.7	4.86*	0.48
*By change in number of standard drinks per day*		29.2	57.9	6.03*	0.62
Can’t recall any	23.5				
Mental health	13.9				
Coughing into elbow	9.6				
**Personal changes in behaviour (353)**					
No change in behaviour	38.2				
*By change in stress levels (DASS-21)*		33.3	66.7	4.50*	0.32
Washed hands properly	33.6				
Socially distanced	33.2				
*By change in physical activity*		44.7	21.6	6.46*	0.49
*By change in number of standard drinks per day*		22.7	54.5	7.77*	0.72
Used sanitizer	18.2				
Coughed into elbow	5.0				
**Suggestions for future safety or health promotion campaigns (244)**					
No need for anything different	30.5				
Better access to timely WA specific information	20.0				
More about how to maintain physical, nutritional, and mental well-being	15.2				
*By change in grain consumption*		22.0	0.0	4.66*	0.59
Clearer direction as to when and where to wear masks	12.9				

*Note.* Percentages (%) are based on multiple responses rather than persons. The total number of responses (*n*) for each aspect are provided in brackets. * = *p*-value < 0.05; x^2^ = Chi Square statistic; *d* = Cohen’s D

From this analysis, it seems those who increased their levels of physical activity were more aware of the need to socially distance from others, as were those who reduced their alcohol intake by reducing the number of days they drank or the number of standard drinks they consumed per day. Similarly, more people who reported lower stress levels during the COVID-19 lockdown felt the need to change their behaviours. Of those that observed social distancing, many increased their level of physical activity or reduced their level of alcohol consumption. Of those who suggested campaigns in the advent of a future lockdown, the only sub-group that showed a statistically significant difference to the general respondents were those who increased their grain intake. Within this group, they felt additional information about how to maintain their physical, nutritional, and mental well-being was needed.

[Recalled] *Physical activity one from the government and lifeline doing their thing for mental health. (21 yo female)*

[Behavioural change] *Spatial distancing, Washing hands properly and often, Coughing into elbow, Avoiding contact, Maintaining exercise. (60 yo, unknown)*

[Future information suggestion] *Information for people with respiratory conditions - more information so we don't get anxious. (41 yo female)*

## Discussion

In the present study, the effect of a three-month lockdown, aimed at reducing the spread of COVID-19, on the community’s physical activity, nutrition and mental well-being was examined. The largest impact, as measured by effect size, was the sense of loss of control respondents had over their lives, followed by an increase in loneliness and alcohol consumption (medium effects). Even though the effects were small, significant decrease in physical activity and increase in the utilisation of snack foods and soft drinks were observed. When looking at mental well-being, while there was a medium effect of lockdown on psychological distress (Kessler-10), only a small effect was observed on the levels of self-reported depression and stress (DASS-21), and interestingly, no observed significant change in community anxiety levels. Taken together, for a community that had very little impact from the disease itself when compared to other countries globally, significant negative changes in physical activity, nutrition, mental and lifestyle were observed in residents of Western Australia during lockdown.

The design of this study does not allow comparison of the results obtained between the lockdown and post-lockdown period to those of a ‘normal’ baseline period. To address this issue, results from the current study were compared to results from the Western Australian Department of Health’s *Health and Wellbeing Surveillance System* (HWSS) from which many of the questions in the current study were drawn [18]. In recent HWSS reports [28, 29] survey responses obtained during March – May 2020 were compared to responses from the same three-month period during 2015–2019 (baseline). Although there were differences in the age profiles of the HWSS and the current study, the findings were similar during lockdown for fruit and vegetable consumption, physical activity, alcohol consumption, psychological distress, and level of control over one’s life. This suggested that the data obtained during the post-lockdown period of the current study does reflect the community norms. Examination of the HWSS data for the full 2019 period suggests however that while physical activity, fruit, vegetable, and alcohol consumption had returned to pre-COVID levels, the sense of control over one’s life and level of psychological distress had not fully returned to their pre-COVID levels [28]. The delayed return of mental well-being to pre-COVID levels may not be surprising given the continued state of awareness of the disease around the world and some local restrictive measures that were still in place during the survey period. This observation is in keeping with analysis of calls to a mental health helpline both prior to, during (increased peak) and following full lockdown (heightened from pre-COVID period) [30].

The sense of loneliness during COVID-19 lockdown as observed in the current study is similar to other communities [31–33], and shown to be associated with adverse mental health effects such as depression, anxiety, and stress-related disorders [33]. This may account for some of the increased level of self-reported depression and stress seen in this study, as loneliness has been estimated to contribute up to 18% of depression in a longitudinal study [34]. It remains unclear why anxiety levels were not altered during lockdown in the present study as others have found increases in all three mental health measures using the same instrument in a web-based surveys of the Ecuadorian and Spanish populations [35, 36]. Of note, the COVID-19 related death rate in these countries were 20–100 times greater than in Australia [37]. Increases in depression and anxiety in the population have been shown during COVID-19 and other epidemics [9, 38], and the effects can persist for at least a year [39]. Despite the low level of community infection in Western Australia, this study still identified a negative impact in mental well-being on a sizeable proportion of the community during the COVID-19 lockdown that remained evident several months after most of the restrictions had been lifted.

The relationship between increased levels of loneliness, depression and alcohol consumption have also been reported. For example, a study of over 6500 older adult Americans observed strong associations between experiencing depression, anxiety, or loneliness and increased alcohol consumption in the past week, with a dose–response relationship between overall mental health symptom burden and drinking either more or less in the past week than before the COVID-19 pandemic [40]. While the overall level of alcohol drinking in the current study was similar in both time periods, about a quarter to a third of respondents reported increasing the amount and/or the frequency of consumption during the lockdown period. This was offset by most drinking behaviours remaining unchanged, or in some cases, reducing. Similar findings have been reported in another study where there was an overall slight reduction in alcohol consumption in French speaking Belgians despite almost a third of people reporting increased alcohol intake [41]. Similar zero net change in population level drinking has been reported in other countries [42–44] which suggests there are multiple factors that impact on a person’s drinking behaviour during period of social isolation, including drinking culture [45].

As in many countries, the Western Australian government responded early to the virus outbreak by implementing several restrictions, some of which had a direct bearing on the community’s ability to engage in their normal physical activity routines. These included closure of gyms, social distancing, use of masks and limiting many class-based exercise sessions to one-on-one, and limiting outdoor exercise to 1 h per day. Consequently, the level of physical activity reported in the current survey significantly reduced during the lockdown which is consistent with findings reported from previous studies [46, 47].

Despite the overall reduction in community physical activity levels, many respondents maintained similar levels of physical activity through the lockdown period, with about a third reducing their levels, while 15% increased them relative to the post-lockdown period. Some understanding of this diverse response comes from a small Canadian qualitative study that identified four themes on how the participants perceived COVID-19 lockdown impacted them: (1) Disruption to Daily Routines, (2) Changes in Physical Activity, (3) Balancing Health, and (4) Family Life [48]. Each of these themes encapsulated both positive and negative effects of the pandemic on physical activity with, as an example, some participants modifying their physical activity routines during the pandemic to maintain levels, while others had difficulty adapting and hence decreased their levels of activity. In the current study, similar differences were identified in the way the respondents perceived the impact of the lockdown in their physical activity with many of those who reduced their activity citing they had limited options, fear of becoming infected and loss of motivation over time. On the other hand, those that increased their activity levels reported they had more time to exercise, managed to adapt their routines and believed it would aid their mental health. In addition, our survey respondents noted that many gyms adapted classes to an online platform to retain members and to meet member needs. Interestingly, a Saudi Arabian study showed there was a significant increase in health-related quality of life and reduced psychological distress in adults who were physically active compared with inactive participants regardless of the level of impact of COVID-19 on their lives [49]. This study corroborates the mental health benefits of physical activity during such a pandemic [50] and suggests that finding the right health promotional message to encourage members of the community to maintain or increase their level of physical activity could have positive outcomes on their mental well-being during periods of great challenge.

In keeping with the range of dietary behaviours reported in the current study, a recent review of dietary changes during COVID-19 found that lockdown both negatively and positively impacted dietary practices globally [51]. In addition, a number of respondents also noted that they had selected local restaurant takeaways to support the community and local businesses. Favourable changes in dietary habits included increases in fresh produce, home cooking and reductions in comfort food and alcohol consumption whilst negative diet habits included reduction in fresh produce, an increase in comfort foods including sweets, fried food, snack foods, and processed foods. Negative food habits are associated with poorer lifestyle outcomes including weight gain, mental health issues, and limited physical activity. With reports of a relationship between diet and mental health in adolescents [52] and adults [53], and alcohol consumption and mental health [40], greater efforts are required to address the large number of people who in the current study reported eating less healthy foods and drinking more alcohol.

With the clear interaction between loneliness, physical activity, dietary behaviours and mental well-being, it is interesting to note that the most common health promotion information recalled by the respondents in the current study was limited to hand hygiene and social distancing, with almost a quarter of respondents not recalling any campaign. Alarmingly, almost 40% of people claimed that their behaviours did not change during this period despite seeing a health campaign, although in some cases, this is because they already observed the requirement. While most people did not see the need for any new or additional health promotional requirements in the advent of a subsequent lockdown, one in five people wanted better access to relevant and timely information and only 15% wanted better information about how to improve their levels of physical activity, dietary behaviours and mental well-being. This is at odds with the emerging view of the close relationship between mental well-being, physical activity and dietary behaviours, especially during a pandemic that greatly increases the level of social isolation and high levels of loneliness. The Western Australian Department of Health has a Health Promotion Strategic Plan that outlines a strategy to address obesity, healthy eating, physical activity, tobacco smoking, alcohol consumption and preventable injuries [54]. Despite this, its social marketing campaigns during 2020 focused on COVID-19, sexual health, needle and syringe safety and urgent primary care services [55]. For the COVID-19 campaigns, the key messages pertained to social distancing, hand hygiene and ‘stay at home if sick’. The findings of this study suggest that extending the current health promotion campaigns to include and emphasize the on-going need and benefits of the wider range of topics as outlined in the Department’s Health Promotion Strategic Plan would be effective in encouraging health promoting behaviours.

The findings from this cross-sectional study provided useful insights about changes in daily life and social activities in Western Australia during the COVID-19 lockdown period. In particular, residents reported a decrease in physical activity level, increased loneliness, less control over life and health, poor nutritional intake (snacks and alcoholic drinks) and increased states of depression and stress. The inclusion of open-ended responses provided a deeper understanding of these key changes. However, several limitations were noted. While the study comprised of a large number of respondents, the use of convenience sampling method may have some risk of bias and limit the generalizability of results. The data were collected using an online questionnaire and was therefore inaccessible to people who did not have a device with internet services to complete the survey. Although the study was conducted shortly after the lockdown period, self-reported responses are still subject to recall bias. Finally, the paired response to questions during both the COVID-19 lockdown and post-lockdown period increased the statistical power of the study, but as the study was of cross-sectional design, inference from causal relationships could not be drawn. Given that confinement was undertaken at home, characteristics of the house and surroundings may influence physical, mental and psychosocial health and this would be an interesting aspect to consider in future research.

## Conclusion

An investigation of a 3 month lockdown in 2020 due to COVID-19 on the physical, mental and psychosocial aspects showed significant negative changes in physical activity, nutrition, alcohol and soft drink consumption, mental well-being and psychosocial health in the Western Australian community. While there is obvious need for governments to disseminate information about how individuals can protect themselves from infectious disease such as COVID-19, it is also clear that they need to provide timely and accurate information about the disease in a balanced way to help improve well-being. These programs need to be complimented with effective health promotion strategies directed at adopting or maintaining positive health related behaviours and ongoing evaluation to ensure they are targeted to all sections of the community. Such strategies need to address the challenges of social isolation, lifestyle changes, physical and nutritional habits.

## Supplementary Information

**Additional file 1.** Supplementary file: Questionnaire used to collect data for the COVID-19 study in Western Australia. Supplementary file description: The file can be viewed using adobe acrobat reader.

## Data Availability

All data analyzed during this study are available from the corresponding author on request and subject to appropriate HREC approvals.

## Abbreviations

HREC: Human Resource Ethics Committee
WA-HWSS: Western Australia Health and Wellbeing Surveillance system
DASS-21: Depression Anxiety Stress Scales
K-10: Kessler Psychological Distress Scale (K-10)
