# Supplementary material for: Life in a time of COVID: a mixed method study of the changes in lifestyle, mental and psychosocial health during and after lockdown in Western Australians
Source: BMC Public Health. 2021 Oct 26;21:1947. doi: 10.1186/s12889-021-11971-7 (PMC8547299; doi:10.1186/s12889-021-11971-7)
Supplement: Supplementary file 1 — Additional file 1. Supplementary file: Questionnaire used to collect data for the COVID-19 study in Western Australia. Supplementary file description: The file can be viewed using adobe acrobat reader. [file 12889_2021_11971_MOESM1_ESM.docx]

**DEMOGRAPHICS**

1. What was your age last birthday?
2. Sex
3. Were you born in Australia?
4. Are you of Aboriginal or Torres Strait Islander origin?
5. What is your residential postcode?

**COVID-19 and FLU (Module FLU)**

1. Have you been tested for COVID-19?
2. Did you test positive to COVID-19? [Skip to COV4 if response = No]
3. Do you experience any of the following ongoing issues? (select all that apply)
4. Persistent shortness of breath 2. Fatigue 3. Chest pressure 4. Blood clots 5. Any other symptoms
5. Has anyone in your household tested positive for COVID-19?
6. Has anyone in your household tested positive for COVID-19

**LIFESTYLE – PHYSICAL ACTIVITY OVERALL**

Please answer each item with respect to your **current** behaviours and **during the COVID-19 lockdown** period.

1. How would you rate your physical activity level?
2. How many days were you **physically active** for a total of **at least 30 minutes per day**?

Open-ended questions:

1. Thinking back to COVID-19 lockdown, what would you say has been the biggest difference made to the following:
   1. Your physical activity (in other words, any changes to your physical activity preferences, types of physical activity, physical activity intensity, etc.)? Please describe those changes.
   2. Physical activity purchases? (at home gym equipment, online physical activity programs, let gym membership lapse, etc.).
2. Are there any other comments you would like to make about any changes to your physical activity levels, habits and choices? Please explain what has changed and why you think it has changed during this time.

**LIFESTYLE – SEDENTARY ACTIVITY**

Please answer each item with respect to your **current** behaviours and **during the COVID-19 lockdown** period.

1. How do you/did you usually spend most of your day?
2. Excluding work time, how many hours per week do you/did you spend watching TV or DVDs, or using the computer, iPad or tablet device (for the internet, to play games etc).

Open ended questions:

1. Thinking back to COVID-19 lockdown, what difference did you make to your screen time? In your opinion, why did these occur? If there was no difference, what were the reasons for this? (e.g. Did you purchase more subscription services? Did you watch more movies or TV shows? Keep in touch with family and friends via screens? etc.). Please expand.
2. Thinking back to COVID-19 lockdown, what difference did isolation make to your leisure time? (e.g. Did you engage in any new or existing hobbies? Did you purchase and use more board games, card games, jigsaw puzzles, craft materials etc.). Please expand.

**LIFESTYLE – NUTRITION**

Please answer each item with respect to your **current** behaviours and **during the COVID-19 lockdown** period.

1. How many serves of **vegetables** do/did you **usually eat each day**? A serve of vegetables is equal to half a cup of cooked vegetables or 1 cup of salad.
2. How many serves of **fruit** do/did you **usually eat each day**? A serve of fruit is equal to one medium piece, two small pieces of fruit or one cup of diced fruit.
3. How many serves of **grain (cereal food)** do/did you **usually eat each day**? A serve of grain is equal to 1 slice of bread or ½ cup cooked rice or pasta.
4. How many serves of **lean meat, poultry, fish, eggs, nuts or legumes** do/did you **usually eat each day**? A serve is equal to 65g lean meat, 2 eggs or 1 cup of legumes.
5. How many serves of **milk, yoghurt, cheese or alternatives** do/did you **usually eat each day**? A serve is equal to 1 cup of milk, 2 slices of cheese or ¾ cup of yogurt.
6. On average, **how many days per week** do/did you **usually** drink soft drink, cordials, sports drinks or caffeinated energy drinks? Please do not include diet varieties. [skip to NUT5 if answer =0]
7. How many **cups (250mls)** of soft drink, cordials, sports drinks or caffeinated energy drinks do you **usually drink on each of those days**?
8. On average, **how many times per week**, do/did you have meals or snacks such as pies, burgers, pizza, chicken or chips from places like McDonalds, Hungry Jacks, Pizza Hut or Red Rooster?
9. In a **typical** **week**, **how often** do you /did you usually drink **alcohol**?
10. On a day when you drink alcohol, **how many standard drinks** do you usually have? A standard drink is equivalent to a schooner of low strength or midi of full strength beer, a glass of wine or a nip of spirits.

Open-ended responses:

1. Thinking back to COVID-19 lockdown, what would you say has been the biggest difference you have made to the following:

- Your diet (in other words, any changes to your food preferences, types of food, food preparation, cooking, alcohol intake etc)? Can you describe those changes?
- Dining practices (e.g., less dining out, more take away food, more food deliveries, etc.?) Please explain what has changed and why you think it has changed during this time.

**KESSLER PSYCHOLOGICAL DISTRESS SCALE**

Please answer each item with respect to your behaviours in the **past four weeks** and during a **typical four weeks of the COVID-19 lockdown** period.

1. About how often did you feel tired out for no good reason?
2. About how often did you feel nervous?
3. About how often did you feel so nervous that nothing could calm you down?
4. About how often did you feel hopeless?
5. About how often did you feel restless or fidgety?
6. About how often did you feel so restless you could not sit still?
7. About how often did you feel depressed?
8. About how often did you feel everything was an effort?
9. About how often did you feel so sad that nothing could cheer you up?
10. About how often did you feel worthless?
11. How many days were you TOTALLY UNABLE to work, study or manage your day to day activities because of these feelings?
12. [Aside from those days], How many days were you ABLE to work or study or manage your day to day activities, but had to CUT DOWN on what you did because of these feelings? ,
13. How many times have you seen a doctor or any other health professional about these feelings?
14. How often have physical health problems been the main cause of these feelings?

**DEPRESSION, ANXIETY STRESS SCALE**

Please answer each item with respect to your behaviours over the **past week** and during a **typical week of the COVID-19 lockdown** period.

1. I found it hard to wind down
2. I was aware of dryness in my mouth
3. I could not seem to experience any positive feelings at all
4. I experienced breathing difficulty (e.g., excessive rapid breathing, breathlessness in the absence of physical exertion)
5. I found it difficult to work up the initiative to do things
6. I tended to over-react to situations
7. I experienced trembling (e.g., in the hands)
8. I felt I was using a lot of nervous energy
9. I was worried about situations I might panic and make a fool of myself
10. I felt I had nothing to look forward to
11. I found myself getting agitated
12. I found it difficult to relax
13. I felt downhearted and blue
14. I was intolerant of anything that kept me from getting on with what I was doing
15. I felt close to panic
16. I was unable to become enthusiastic about anything
17. I felt I wasn’t worth much as a person
18. I felt I was rather touchy
19. I was aware of the action of my heart in the absence of physical exertion (eg sense of heart rate, heart missing a beat)
20. I felt scared without any good reason
21. I felt life was meaningless

**EQ-5D5L**

Under each heading, please SELECT the ONE that best describes your health TODAY

**Mobility**

- I have no problems in walking about
- I have slight problems in walking about
- I have moderate problems in walking about
- I have severe problems in walking about
- I am unable to walk about

**Self-care**

- I have no problems washing or dressing myself
- I have slight problems washing or dressing myself
- I have moderate problems washing or dressing myself
- I have severe problems washing or dressing myself
- I am unable to wash or dress myself

**Usual activities** (e.g. work, study, housework, family or leisure activities)

- I have no problems doing my usual activities
- I have slight problems doing my usual activities
- I have moderate problems doing my usual activities
- I have severe problems doing my usual activities
- I am unable to do my usual activities

**Pain / Discomfort**

- I have no pain or discomfort
- I have slight pain or discomfort
- I have moderate pain or discomfort
- I have severe pain or discomfort
- I have extreme pain or discomfort

**Anxiety / Depression**

- I am not anxious or depressed
- I am slightly anxious or depressed
- I am moderately anxious or depressed
- I am severely anxious or depressed
- I am extremely anxious or depressed

**MENTAL HEALTH CONDITIONS**

Are you currently receiving treatment for:

- - Anxiety yes no,
  - Depression yes no,
  - Stress-related problems yes no
  - Any other mental health problem yes no

[Skip if response = No] Please indicate the start period:

- Before COVID-19 lockdown period
- During COVID-19 lockdown period
- After COVID-19 lockdown period

Open ended questions:

1. Describe what things may have affected (positively and/or negatively) your mental well-being during the COVID-19 lockdown period?

PERCEIVED LACK OF CONTROL (Module LAC)

Please answer each item with respect to your behaviours over the **past four weeks** and during a **typical four weeks period of the COVID-19 lockdown**.

1. How much of the time did you feel a lack of control over your life in general?
2. How much of the time did you feel a lack of control over your personal life?
3. How much of the time did you feel a lack of control over your health?

Open ended questions:

1. Describe what things may have affected (positively and/or negatively) your feelings of control during the COVID-19 lockdown period?
2. We would like to know how good or bad your health is TODAY. On a scale of 0 – 100 where 100 is the best health you can imagine and 0 is the worst health you can imagine, what is the number that indicates your health TODAY.

**LONELINESS**

Please answer each item with respect to your **current** behaviours and **during the COVID-19 lockdown** period.

1. How often do/did you feel that you lack companionship?
2. How often do/did you feel left out?
3. How often do/did you feel isolated from others?

**SOCIAL CAPITAL**

1. Under normal circumstances, how many groups/associations do you belong to? Include church groups, social groups, sporting groups, political groups, professional groups etc.

Open ended questions:

1. Thinking back to COVID-19 lockdown, how would you rate your ability to participate in your groups/associations: (1. Less involved than normal, 2 about the same, 3 more involved than normal).
2. How did the COVID-19 lockdown period make a difference to the level of social connectedness that you would usually experience. Please explain further (e.g. What were the differences? In your opinion, why did these occur? What impact did it have on your overall well-being? etc.).

**FAMILY FUNCTIONING**

Thinking back to your usual circumstances **pre COVID-19 lockdown**, please answer the following questions about family functioning.

1. We usually don’t get on well together.
2. Planning family activities is usually difficult.
3. We usually avoid discussing our fears and concerns openly with each other.
4. Making decisions is usually a problem in our family because we misunderstand each other.
5. During the COVID-19 lockdown period, family functioning was generally

1. Better than normal, 2. About the same, 3. Worse than normal.

**BRIEF RESILIENCE SCALE**

Please indicate the extent to which you agree with each of the following statements:

1. I tend to bounce back quickly after hard times
2. I have a hard time making it through stressful events
3. It does not take me long to recover from a stressful event
4. It is hard for me to snap back when something bad happens (
5. I usually come through difficult times with little trouble
6. I tend to take a long time to get over set-backs in my life

**LIFESTYLE – TOBACCO SMOKING**

Please answer each item with respect to your **current** behaviours and **during the COVID-19 lockdown** period.

1. Which of the following best describes your smoking status prior to COVID-19 lockdown? This includes cigarettes, e-cigarettes, cigars and pipes.
2. During COVID-19 lockdown period, how would you describe your tobacco use? (1 less than normal, 2 about the same, 3 more than normal; 4 quit; 5 NA don’t smoke).
3. Which of the following best describes your current smoking status?

**RECALL OF NOTICED HEALTH PROMOTION CAMPAIGNS**

1. Please list the topic of any health promotion campaigns you recall that ran during COVID-19 lockdown.
2. Please describe any changes you made to your behaviours as a result of these campaigns
3. What health promotion messages should have been provided to the community during COVID-19 lockdown that were missing at the time?

**LIFESTYLE - BODY MEASUREMENTS**

1. What is your height without shoes? ___metres
2. How much do you weigh without clothes or shoes?
3. Currently ___kg
4. Before COVID-19 lockdown _____kg

**SOCIAL CHARACTERISTICS**

1. What is the highest qualification you have completed?
   - Post graduate qualifications (Masters or doctoral)
   - Bachelor degree
   - Diploma or certificate or trade/apprenticeship
   - Completed high school
   - Did not complete high school
2. Which ONE of the following best describes your employment status? Are you:
3. Currently b. During COVID-19 lockdown

- Self employed
  - Are you receiving Job Keeper No/Yes
- Employed for wages, salary or payment-in-kind
  - Are you receiving Job Keeper No/Yes
- Unemployed for less than one year
  - Are you receiving Job Seeker No/Yes
- Unemployed for more than one year
  - Are you receiving Job Seeker No/Yes
- Engaged in home duties
- Retired
- Unable to work
- A student
- Other

1. Do you do some form of work that takes you away from home for a set period each week or month? [For example, work fly-in fly-out or similar arrangement]
2. During the COVID-19 lockdown, did you do some form of work that took you away from home for a set period each week or month? [For example, work fly-in fly-out or similar arrangement]
3. What best describes your living arrangements?
4. Currently b. During COVID-19 lockdown

- Living with my parent(s)
- Living with other family members
- Living with friends
- Living with a partner/spouse and children
- Living with a partner/spouse but no children
- Living with no partner/spouse but with children
- Living alone
- Living in a nursing home
- Living in a retirement village
- Other living arrangements

1. Which best describes your household money situation?
2. Currently b. During COVID-19 lockdown

- I am / we are spending more money than I / we get
- I / we have just enough money to get us through to the next pay day
- There’s some money left over but I / we just spend it
- I / we can save a bit every now and then
- I / we can save regularly
- I / we can save a lot

1. I would now like to ask you about your household's income. We are interested in how income relates to health, lifestyle and access to health services. Before tax is taken out, which of the following ranges best describes your household's income, from all sources, over the past 12 months?

- Under $20,000
- $20,000 - $40,000
- $40,000 - $60,000
- $60,000 - $80,000
- $80,000 - $100,000
- $100,000 - $120,000
- $120,000 - $140,000
- $140,000 - $160,000
- More than $160,000
- Prefer to not answer

**OPEN QUESTIONS**

1. Provide examples of how you and your family were **positively** affected during the COVID-19 lockdown.
2. Provide examples of how you and your family were **negatively** affected during the COVID-19 lockdown.
3. Thinking about the impact of COVID-19 on physical and mental well-being, are there any other comments you would like to make?
